# Resuscitation of very preterm infants with 30% vs. 65% oxygen at birth: study protocol for a randomized controlled trial

**DOI:** 10.1186/1745-6215-13-65

**Published:** 2012-05-23

**Authors:** Denise Rook, Henk Schierbeek, Anne C van der Eijk, Mariangela Longini, Giuseppe Buonocore, Maximo Vento, Johannes B van Goudoever, Marijn J Vermeulen

**Affiliations:** 1 Department of Pediatrics, Division of Neonatology, Erasmus Medical Center - Sophia Children’s Hospital, Rotterdam, The Netherlands; 2 Department of BioMechanical Engineering, Faculty of Mechanical, Maritime & Materials Engineering, Delft University of Technology, Delft, The Netherlands; 3 Department of Pediatrics, Obstetrics and Reproductive Medicine, University of Siena, Siena, Italy; 4 Department of Pediatrics, Division of Neonatology, University Children’s Hospital La Fe, Valencia, Spain; 5 Department of Pediatrics, Emma Children’s Hospital - AMC, Amsterdam, The Netherlands; 6 Department of Pediatrics, VU University Medical Center, Amsterdam, The Netherlands

**Keywords:** Preterm infants, Resuscitation, Oxygen, Bronchopulmonary dysplasia, Oxidative stress, Glutathione synthesis

## Abstract

**Background:**

Resuscitation at birth with 100% oxygen is known to increase the oxidative burden with concomitant deleterious effects. Although fractions of inspired oxygen (FiO_2_) < 100% are widely used in preterm infants, starting resuscitation at a (too) low FiO_2_ may result in hypoxia. The objective of this study is to compare the safety and efficacy of resuscitating very preterm infants with an initial FiO_2_ of 30% versus 65%.

**Methods/design:**

In this double-blind, randomized controlled trial, 200 very preterm infants with a gestational age < 32 weeks will be randomized to start resuscitation after birth with either 30% or 65% oxygen. The FiO_2_ will be adjusted based on oxygen saturation measured by pulse oximetry (SpO_2_) and pulse rate (which should be over 100 beats per minute) in order to achieve a target SpO_2_ of 88–94% at 10 min of life. The FiO_2_ and pulse oximetry data will be continuously recorded.

The primary outcome is survival without bronchopulmonary dysplasia, as assessed by a physiological test at 36 weeks postmenstrual age. The secondary outcomes include the time to achieve SpO_2_ > 88%, Apgar score at 5 min, cumulative O_2_ exposure, oxidative stress (as determined by glutathione synthesis and oxidative stress markers), retinopathy of prematurity, brain injury and neurodevelopmental outcome at 2 years of age.

This study will provide insight into determining the appropriate initial FiO_2_ to start resuscitation of very preterm infants.

**Trial registration:**

http://www.trialregister.nl, NTR243.

## Background

Resuscitating the newborn at birth with 100% oxygen is known to increase the oxidative burden with concomitant deleterious effects [1]. The latest International Liaison Committee on Resuscitation (ILCOR) guidelines recommend that “for babies born at term it is best to begin resuscitation with air rather than 100% oxygen” and that “administration of supplementary oxygen should be guided by oximetry” [2]. However, for preterm infants, the optimal fraction of inspired oxygen (FiO_2_) to start resuscitation is still unknown. The ILCOR states that “blended oxygen and air may be given judiciously” and “both hyperoxemia and hypoxemia” should be avoided [2].

Several small studies on FiO_2_ for resuscitating preterm infants have been performed. Wang et al. compared the use of initiating the resuscitation of preterm infants with either room air (*n* = 18) or 100% oxygen (*n* = 23) [3]. All infants in the room air group required an increase of the FiO_2_ to achieve the targeted oxygen saturation (SpO_2_), and the authors recommended that room air should not be used for resuscitating preterm infants. Escrig et al. compared initiating resuscitation of preterm infants with a gestational age (GA) ≤ 28 weeks with either 30% or 90% oxygen [4]. In this study, the FiO_2_ in the low-oxygen group (*n* = 19) was increased stepwise to 45%, and the FiO_2_ in the high-oxygen group (*n* = 23) was reduced to 45% to reach the target SpO_2_. In a similar study by Vento et al., resuscitation with 30% oxygen (*n* = 37) resulted in decreased oxidative stress markers and a decreased risk of bronchopulmonary dysplasia (BPD) compared to starting resuscitation with 90% (*n* = 41) [5]. Also in this study, FiO_2_ in both groups was increased stepwise in the low-oxygen group and decreased in the high-oxygen group, reaching 55% at 5 min in both groups.

From these data, it can be concluded that initiating the resuscitation of preterm infants with room air is too low, while starting with 90% FiO_2_ is too high. Because it is important to avoid both hypoxia and hyperoxia, the optimal initial FiO_2_ for resuscitating preterm infants needs to be determined. Therefore, we hypothesize that resuscitation of very preterm infants (GA < 32 weeks) with an initial FiO_2_ of 30% is safe, decreases oxidative stress and improves outcome compared to resuscitation with an initial FiO_2_ of 65%.

## Methods/design

### Trial design

The study is a double-blind, randomized controlled trial and will be performed in the Neonatal Intensive Care Unit (NICU) of the Erasmus MC - Sophia Children’s Hospital, Rotterdam, The Netherlands. The study is investigator-initiated, without funding from the pharmaceutical industry. The study protocol has been approved by the Erasmus MC Medical Ethics Committee. Serious adverse events (death, retinopathy of prematurity (ROP) ≥ grade 3 and intraventricular hemorrhage (IVH) ≥ grade 3) will be reported to the medical ethics committee, which will monitor the study safety.

### Subjects

The inclusion criteria are infants with a GA < 32 weeks born at the Erasmus MC-Sophia Children’s Hospital. Assessment of the GA will be based on early fetal ultrasonography or on the date of the last menstrual period. The exclusion criteria are any known major congenital malformations, chromosome defects, or metabolic, endocrine or renal disorders.

Because this study involves an acute intervention at birth, written informed consent will be obtained antenatally. All mothers admitted to Erasmus MC-Sophia Children’s Hospital at risk for preterm delivery before 32 weeks of gestation (e.g., premature labor, preeclampsia, intrauterine growth retardation) will be approached for participation in the study. When parents have consented to participate in the study and there has been an actual preterm delivery, the preterm infant will be included at birth.

### Research setting and randomization

**Figure 1:**
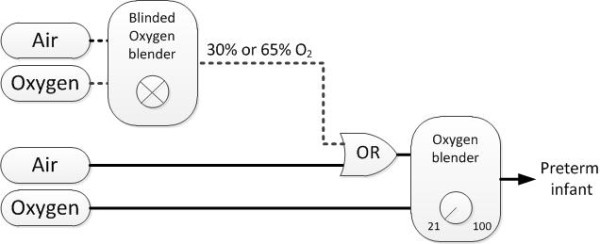
**Adjusted resuscitation unit.*** Dotted lines* depict the adjustments made to the resuscitation unit. The blinded oxygen blender is randomized to administer either 30% or 65% oxygen. The OR port depicts the switch by which the research setting can be activated or deactivated.

The Erasmus MC-Sophia Children’s Hospital has six resuscitation units, which have been modified for this study by adding an additional oxygen blender (PM5200, Precision Medical Inc., Northampton, PA, USA) (Figure 1). This additional oxygen blender is not visible to the physician and will be randomized after each inclusion to either 30% or 65% oxygen using a computer-generated list.

When an infant with prenatal consent is born, the physician will activate the research setting by activation of a switch just before delivery. By activating this research switch, the regular oxygen blender (Bird Ultrablender, Cardinal Health, Dublin, OH, USA) will be connected to the additional oxygen blender. Administered oxygen will come from the additional oxygen blender, randomized to 30% or 65% oxygen, and thus resuscitation will be started with either 30% or 65% oxygen.

### Resuscitation

All resuscitations of preterm infants are performed by a neonatologist or an experienced neonatologist in training. Immediately after cord clamping, the infant will be placed on the resuscitation unit. The resuscitation is performed according to standard guidelines, i.e., the infant is stimulated and heat loss is prevented. A disposable SpO_2_ sensor (Nellcor Max-N, Covidien, Dublin, Ireland) will be applied to the right hand or wrist before switching on the pulse oximeter (Nellcor OxiMax N-600x, Covidien, Dublin, Ireland). Infants will be resuscitated with either a flow inflating mask (Jackson Reese modification T-piece system breathing system, Intersurgical, Wokingham, UK) or a T-piece resuscitator (Neopuff, Fisher & Paykel Healthcare, Auckland, New Zealand), according to the physician’s preferences.

Resuscitation is started with either 30% or 65% oxygen, for which the physician will be blinded. The objective of the resuscitation is to achieve a target SpO_2_ of 88–94% at 10 min after birth. If the pulse rate remains stable and over 100 beats per minute (bpm), no adjustment of FiO_2_ is advised. At all times, the physician can adjust the FiO_2_ if the clinical situation is not satisfactory (e.g., persistent bradycardia or SpO_2_ >94%). To adjust the FiO_2_, the physician deactivates the research switch by which the additional oxygen blender is deactivated, and the administered FiO_2_ is supplied via the regular oxygen blender. The FiO_2_ can then be manually adjusted to the desired FiO_2_, without the physician being aware of the initial FiO_2_ to which the infant was randomized.

### Outcome parameters

#### Primary outcome

Survival without BPD at 36 weeks postmenstrual age (PMA).

#### Secondary outcomes

1. Resuscitation: Apgar score at 5 min, time after birth to achieve SpO_2_ > 88% and cumulative O_2_ exposure during resuscitation*.*

2. Oxidative stress: Glutathione (GSH) synthesis and oxidative stress markers

3. Incidence of ROP and brain injury.

4. Neurodevelopmental outcome at 2 years of age.

**Figure 2:**
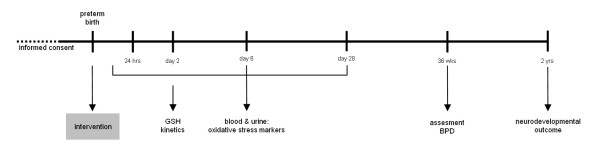
**Timeline of the study design.*** GSH* = glutathione, *BPD* = bronchopulmonary dysplasia.

The timeline of the study design is depicted in Figure 2.

### Collection resuscitation data

Medical record documentation often varies from actual interventions, especially in acute situations [6]. Therefore, video recordings will be made to analyze resuscitation with regard to time of birth and the timeline of the resuscitation (e.g., time to start SaO_2_ recording, time to start respiratory support and the time to intubation, when applicable). The time of birth is defined as the time of cord clamping. The FiO_2_ will be continuously recorded (1 Hz) using a medical oxygen monitor (MX300 Medical Oxygen Monitor, Teledyne, City of Industry, CA, USA). The SpO_2_ will be continuously recorded (0.5 Hz) using serial port reader software (TeraTerm, Open Source Software).

### Clinical definitions

#### Bronchopulmonary dysplasia

Two definitions for the diagnosis of BPD will be used. First, the definition of BPD for preterm infants with a GA < 32 weeks described by Jobe et al. will be used (Table 1) [7]. According to this definition, BPD is present when an infant is treated with FiO_2_ > 21% for at least 28 days and is further classified according to the need for oxygen and/or respiratory support at 36 weeks PMA.

**Table 1:** Criteria for bronchopulmonary dysplasia for preterm infants with a gestational age < 32 weeks

Treatment with oxygen >21% for at least 28 days **plus**		
Mild BPD		Breathing room air at 36 weeks PMA
Moderate BPD		Need for < 30% oxygen at 36 weeks PMA
Severe BPD		Need for ≥ 30% oxygen and/or positive pressure at 36 weeks PMA

Second, the diagnosis of BPD will be assessed at 36 weeks PMA based on the physiological criteria of Walsh et al. [8]. Infants treated with mechanical ventilation or continuous positive airway pressure (CPAP), or infants receiving FiO_2_ ≥ 30% oxygen with oxygen saturations < 96% are diagnosed with BPD. For infants receiving < 30% oxygen or infants receiving ≥ 30% oxygen with oxygen saturations > 96%, a timed oxygen reduction test will be performed as described by Walsh et al. [8]. During the timed oxygen reduction test, BPD is diagnosed when oxygen saturations are < 90% for more than 5 consecutive min or < 80% for more than 15 s. No BPD is defined as oxygen saturations ≥ 90% during weaning to room air.

#### Retinopathy of prematurity

As part of standard care, ROP will be assessed at a postnatal age of 5 weeks by a pediatric ophthalmologist. ROP will be diagnosed and classified according to the International Classification of Retinopathy of Prematurity (ICROP) [9].

#### Brain injury

Substantial brain injury will be diagnosed according to the definition previously described in the EUNO trial [10], i.e., grade 3 or 4 IVH [11] or periventricular leukomalacia based on ultrasound images and/or MRI of the brain [12].

#### Neurodevelopmental outcome

As part of standard care, all preterm infants born with a GA < 32 weeks will be followed until 2 years of age. At 2 years, certified neurodevelopmental physiologists will evaluate the infants using the Bayley Scales of Infant Development, 3rd Edition (BSID III).

### Glutathione concentration and synthesis rates

#### Tracer infusion protocol and blood sampling

On the 2nd postnatal day, a primed (20 μmol·kg^−1^) continuous (20 μmol·kg^−1^·d^−1^) infusion of [U-^13^ C]glycine (99% enriched, Cambridge Isotope Laboratories, Andover, MA, USA; sterility and pyrogenicity tested) will be administered for 8 h using a Perfusor fm infusion pump (B|Braun Medical B.V., Oss, The Netherlands). Blood will be sampled from an indwelling arterial catheter after 6, 7 and 8 h, and collected in EDTA-containing microtainers. After centrifugation at 3,500 × g for 10 min, the plasma fraction will be removed, and the lower layer (containing primarily erythrocytes) will be reconstituted to its original volume with ice-cold distilled water. To calculate the fractional synthesis rates (FSR) and absolute synthesis rates (ASR) of GSH, concentration and enrichment of GSH and its precursor glycine will be determined in the erythrocytes.

#### Glutathione enrichment and concentration

Analysis of GSH will be performed on a LC-Isolink interface coupled to a Delta XP isotope ratio mass spectrometer (LC-IRMS) (Thermo Fisher, Bremen, Germany) using a recently developed method [13]. This highly sensitive method requires only a very small sample and does not require derivatization of the sample.

#### Glycine enrichments

The erythrocyte enrichment of ^13^C glycine will be measured by gas chromatography-mass spectrometry (GCMS) as its ethyl chloroformate (ECF) ester derivatives, using a MSD 5975 C Agilent GCMS (Agilent Technologies, Amstelveen, The Netherlands). Briefly, 25-μl aliquots of the remaining supernatant used for the GSH analysis will be acidified by adding 50 μl of 0.1 M HCl and diluted with 125 μl of distilled water. ECF derivatization of the samples will be performed according to a modified procedure of Hušek [14]. A CP-Sil 17 column (25 m × 0.25 mm id, 0.12-μm film thickness; Varian, Middelburg, The Netherlands) will be used for the separation. The samples will be measured using a selected ion monitoring mode (SIM) method. The mass fragments with a mass to charge (*m/z*) of 102.1 for unenriched (*M*)and an *m/z* 103.1 for the enriched (*M* + 1) glycine, respectively, have been selected for this purpose.

#### Calculations

The FSR_GSH_ represents the fraction of the total intraerythrocytic GSH pool that is renewed per unit of time and is expressed as %/d.

(1) $${\mathrm{FSR}}_{\mathrm{GSH}}(\%/d)=\frac{slope\;E_{[U{-}^{13}C]GSH_{t6,7,8}}}{E_{intraerythrocytic[U{-}^{13}C]glycine}}\times 24h\times 100\%\text{,}\;$$

where E stands for the enrichment expressed as MPE. The nominator (product) of this equation represents the hourly increase in [U-^13^ C]glycine bound to GSH, as calculated from the increase in enrichment between 6 and 8 h of infusion. The denominator (precursor) represents the intraerythrocytic U-^13^ C enrichment of free glycine at isotopic steady state.

Subsequently, the intravascular ASR_GSH_, which represents the absolute amount of GSH that is produced per unit of time (mg/(kg·day)), can be calculated using the following equation:

(2) $${\mathrm{ASR}}_{\mathrm{GSH}}(\text{mg}/(\text{kg}\times \text{d})={\mathrm{FSR}}_{\mathrm{GSH}}/(100\text{ × conc}.\text{ × }307\text{ × ht × }0.075)$$

where conc. is GSH concentration in mmol/l packed erythrocytes, 307 is the molecular weight of GSH, ht is hematocrit, and 0.075 is the estimated circulating volume in a preterm neonate, expressed as l/kg.

### Oxidative stress markers

Oxidative stress markers in plasma and urine will be determined within 24 h of birth, on postnatal day 6 and on postnatal day 28.

#### Non-protein bound iron (NPBI)

Blood samples, drawn from an arterial catheter or via a heel prick, will be collected in heparinized microtainers and immediately placed on melting ice. After centrifugation at 3,500 × g for 10 min, the plasma fraction will be removed from the lower layer and stored at −80 °C until further analysis. Plasma samples will be shipped on dry ice to the University of Siena (Siena, Italy), where the NPBI will be determined according to previously described methods [15].

#### Isoprostanes and 8-hydroxy-2’-deoxyguanosine (8-oxo-dG)

Urine will be collected by placing gauze in the infants’ diapers. After centrifugation at 2,800 × g for 5 min, the urine will be stored at −80 °C until further analysis. Urine samples will be shipped on dry ice to the University Hospital LA Fe (Valencia, Spain), where the urinary isoprostane and 8-oxo-dG will be determined according to previously described methods [16].

### Statistical analysis

Power calculation based on the incidence of BPD shows that, with an incidence of 30% and an expected reduction of 15%, 100 infants per group will be needed to find a statistically significant difference with an α of 0.05 and a power of 0.80. Differences between groups will be assessed using the Mann-Whitney test for continuous measurements and the chi-square test for categorical measurements (*p* < 0.05).

## Discussion

Although this study concerns the acute intervention of neonatal resuscitation, the randomization and blinding are optimal in this study design. Because cases of acute preterm delivery will not be included in this study, the main limitation will be the selection bias. Since informed consent will be obtained before birth, only mothers who are actually hospitalized antenatally will be approached by the researchers. Consequently, all included infants will have received at least one dose of prenatal steroids. As prenatal steroids have proven to be beneficial to immature lungs, included infants will likely have fewer respiratory difficulties than the acute cases. Furthermore, administration of prenatal steroids is associated with increased antioxidant enzyme activity, which reduces susceptibility to hypoxia and to oxidative damage as a result of hyperoxia [17]. In short, the studied cases may show less morbidity such as BPD than the total population of very preterm infants.

The selection bias could be circumvented by a waiver of informed consent. In 1996, the Food and Drug Administration (FDA) and the Department of Health and Human Services (DHHS) published guidelines on exceptions from the informed consent requirements in specific situations [18]. For emergency research, these guidelines stipulate that the institutional review board (IRB) may approve a clinical investigation without requiring informed consent from all research subjects after meeting certain criteria. These criteria include informed consent not being feasible because the intervention must be performed before consent from the subjects’ legally authorized representative can be obtained, as would be the case in this study when preterm infants are born acutely. Because informed consent can be sought in a sufficient number of very preterm deliveries, we decided that it would not be ethical to use a waiver of informed consent.

This study is performed in an affluent health care setting, and blended oxygen might not be available in all hospitals. However, according to international guidelines, preterm infants should be born in a tertiary hospital whenever possible, and, in Western countries, most very preterm infants are indeed born within a tertiary hospital. Since it has been shown that room air and 100% oxygen are both not ideal in the resuscitation of preterm infants, it is important to study the optimal FiO_2_ to start resuscitation of these infants.

## Trial status

The trial is currently enrolling patients. We expect to finish patient recruitment in March 2012 and present the results over the course of 2012.

## Abbreviations

FiO2, fraction of inspired oxygen; SpO2, oxygen saturation; GA, gestational age; BPD, bronchopulmonary dysplasia; PMA, postmenstrual age; GSH, glutathione.

## Competing interests

The authors declare that there are no competing interests

## Authors’ contribution

DR: study design, acquisition of data, analysis and interpretation of data, drafting and revising the manuscript. HS, ML, GB, MV: analysis and interpretation of data, revising the manuscript. AvdE: technical support for design and data acquisition, revising the manuscript. JvG: conception and study design, interpretation of data, revising the manuscript. MJV: conception and study design, interpretation of data, revising the manuscript. All authors have read and approved the final manuscript.

## References

[B1] Saugstad OD Ramji S Soll RF Vento M Resuscitation of Newborn Infants with 21 % or 100 % Oxygen: An Updated Systematic Review and Meta-Analysis Neonatology 2008 94 176 182 10.1159/000143397 18612215

[B2] Perlman JM Wyllie J Kattwinkel J Atkins DL Chameides L Goldsmith JP Guinsburg R Hazinski MF Morley C Richmond S Part 11: neonatal resuscitation: 2010 International Consensus on Cardiopulmonary Resuscitation and Emergency Cardiovascular Care Science With Treatment Recommendations Circulation 2010 122 S516 S538 10.1161/CIRCULATIONAHA.110.971127 20956259

[B3] Wang CL Anderson C Leone TA Rich W Govindaswami B Finer NN Resuscitation of preterm neonates by using room air or 100 % oxygen Pediatrics 2008 121 1083 1089 10.1542/peds.2007-1460 18519476

[B4] Escrig R Arruza L Izquierdo I Villar G Saenz P Gimeno A Moro M Vento M Achievement of targeted saturation values in extremely low gestational age neonates resuscitated with low or high oxygen concentrations: a prospective, randomized trial Pediatrics 2008 121 875 881 10.1542/peds.2007-1984 18450889

[B5] Vento M Moro M Escrig R Arruza L Villar G Izquierdo I Roberts LJ Arduini A Escobar JJ Sastre J Asensi MA Preterm Resuscitation With Low Oxygen Causes Less Oxidative Stress, Inflammation, and Chronic Lung Disease Pediatrics 2009 124 e439 e449 10.1542/peds.2009-0434 19661049

[B6] Rich WD Leone T Finer NN Delivery room intervention: improving the outcome Clin Perinatol 2010 37 189 202 10.1016/j.clp.2010.01.011 20363455

[B7] Jobe AH Bancalari E Bronchopulmonary dysplasia Am J Respir Crit Care Med 2001 163 1723 1729 11401896 10.1164/ajrccm.163.7.2011060

[B8] Walsh MC Yao Q Gettner P Hale E Collins M Hensman A Everette R Peters N Miller N Muran G Impact of a physiologic definition on bronchopulmonary dysplasia rates Pediatrics 2004 114 1305 1311 10.1542/peds.2004-0204 15520112

[B9] The International Classification of Retinopathy of Prematurity revisited Arch Ophthalmol 2005 123 991 999 16009843 10.1001/archopht.123.7.991

[B10] Mercier JC Hummler H Durrmeyer X Sanchez-Luna M Carnielli V Field D Greenough A Van Overmeire B Jonsson B Hallman M Baldassarre J Inhaled nitric oxide for prevention of bronchopulmonary dysplasia in premature babies (EUNO): a randomised controlled trial Lancet 2010 376 346 354 10.1016/S0140-6736(10)60664-2 20655106

[B11] Papile LA Burstein J Burstein R Koffler H Incidence and evolution of subependymal and intraventricular hemorrhage: a study of infants with birth weights less than 1,500 gm J Pediatr 1978 92 529 534 10.1016/S0022-3476(78)80282-0 305471

[B12] De Vries LS Regev R Pennock JM Wigglesworth JS Dubowitz LM Ultrasound evolution and later outcome of infants with periventricular densities Early Hum Dev 1988 16 225 233 10.1016/0378-3782(88)90103-X 3288470

[B13] Schierbeek H Te Braake F Godin JP Fay LB van Goudoever JB Novel method for measurement of glutathione kinetics in neonates using liquid chromatography coupled to isotope ratio mass spectrometry Rapid Commun Mass Spectrom 2007 21 2805 2812 10.1002/rcm.3148 17661340

[B14] Husek P Rapid derivatization and gas chromatographic determination of amino acids J Chromatogr 1991 552 289 299

[B15] Paffetti P Perrone S Longini M Ferrari A Tanganelli D Marzocchi B Buonocore G Non-protein-bound iron detection in small samples of biological fluids and tissues Biol Trace Elem Res 2006 112 221 232 10.1385/BTER:112:3:221 17057261

[B16] Milne GL Sanchez SC Musiek ES Morrow JD Quantification of F2-isoprostanes as a biomarker of oxidative stress Nat Protoc 2007 2 221 226 10.1038/nprot.2006.375 17401357

[B17] Vento M Aguar M Escobar JJ Arduini A Escrig R Brugada M Izquierdo I Asensi MA Sastre J Saenz P Gimeno A Antenatal Steroids and Antioxidant Enzyme Activity in Preterm Infants: Influence of Gender and Timing Antioxid Redox Signal 2009 11 12 2945 2955 10.1089/ars.2009.2671 19645572

[B18] Protection of human subjects http://www.access.gpo.gov/nara/cfr/waisidx_02/21cfr50_02.html

